# Are Spanish Surveys Ready to Detect the Social Factors of Obesity?

**DOI:** 10.3390/ijerph191811156

**Published:** 2022-09-06

**Authors:** Cecilia Díaz-Méndez, Sonia Otero-Estévez, Sandra Sánchez-Sánchez

**Affiliations:** Department of Sociology, University of Oviedo, 33006 Oviedo, Spain

**Keywords:** food surveys, food change, food policies, sociology of food, obesity

## Abstract

The social origins of obesity are now recognised: a problem that is initially biological is today a public health problem with a social origin. This paper raises the question of whether the official statistical sources used to understand changes in diet are able to detect this shift in analysis. After reviewing the social factors that explain obesity, we examine the official Spanish statistics that can inform about dietary changes: the ENS National Health Survey, the EPF Family Budget Survey, and the EET Time Use Survey, all carried out by the Spanish Statistical Office. All of them include socio-demographic variables and some locational variables. However, the lack of health variables in the economic survey and the lack of social variables in the health survey prevent the gathering of reliable scientific evidence to offer solid support in stopping the obesity epidemic. Food has become particularly important as one of the main areas where unhealthy decisions and choices involve high risk; the situation also demonstrates the relationship between social inequality and obesity. Obesity is now understood in a radically different way and the origin of the problem lies in social and cultural factors. The current surveys do not provide the resources to capture the social causality of obesity, but slight modifications would help expand their capabilities and offer reliable scientific evidence to stop the obesity epidemic.

## 1. Introduction

In recent years, obesity figures have become alarming, both in Spain and elsewhere. Obesity has increased in countries without food shortages, but also in poor countries, where it coexists with hunger [1,2,3,4]. Concern about its spread has prompted in-depth study of its causes from a variety of perspectives, revealing the social origins of a problem that initially seemed strictly biological and individual; these social roots are no longer questioned [5]. Therefore, research into the social factors linked to obesity is a constant in the academic literature, especially since the appearance of Wilkinson and Marmot’s work *Social Determinants of Health* in 1998 [6] and following the World Health Organisation’s report on obesity and overweight as a public health problem [7].

Since the beginning of the 21st century, researchers in the fields of both public health and social science have begun to address this issue and offer explanations about the origins and/or consequences of a problem associated with changes in eating habits and the social transformations of modern life [8,9,10,11,12].

Spain is no stranger to the problem of obesity and, according to the National Health Survey, the proportion of obese adults rose from 2.4% of the population in 1987 to 17.4% in 2017 [13]. Studies showing the social factors linked to obesity point to multiple causes, although they focus on different causal factors. The majority of them associate obesity with the types of products consumed, i.e., obesity is attributed to the poor composition of the diet, and social factors influence the food choices that lead to obesity. Obesity is considered to be caused by dietary choices that take individuals away from healthy eating patterns, and socio-demographic variables explain these choices. This has been confirmed in both older and more recent studies [14,15]. For some, inappropriate choices are prompted by the consumer’s economic means, preventing them from choosing the healthiest products, something that has been observed in a variety of contexts and at different historical moments [16,17,18]; others claim that these incorrect choices are made because some social groups are insufficiently educated about nutrition [19,20]. Many of these studies focus on food spending, an explanation initiated by Drewnoswski and Darmon [21] and today continued in studies on poverty and food [22,23,24].

It is increasingly common to associate obesity with physical activity—or lack of it—and to incorporate this as a variable in analysis [25]. Although the data are not always conclusive and it is not clear that physical exercise reduces obesity in the same way that dietary control does [26], a more sedentary lifestyle has been observed among the obese population; this is the case in Spain, and socio-demographic variables continue to influence this behaviour [15]. Studies have been extended to include so-called obesogenic environments, understood as those that promote unhealthy lifestyles, and they incorporate locational variables into the analysis of obesity [27,28].

There is also a group of studies dedicated to exploring the effects of the pressure exerted by society on individuals to adopt behaviours and roles that condition their eating habits. Behind these explanations are variables relating to motivation: attitudes, perceptions, beliefs, the meaning attributed to food, and the pressure on people to conform to a body model. They also explain the effect that women’s role in looking after others in the home has on their own eating behaviours and those of their families [29,30]. It has been confirmed that beliefs about health and food, or the significance attributed to foods, determine whether they are included in or excluded from the diet, which may then lead to obesity [8,31,32]. The importance of social relationships connected with food as mediating factors in beliefs and the meanings attributed should also be noted, given that eating is essentially an act of social relationship [33,34,35]. The body shows a person’s social position and their lifestyle, as well as their cultural and economic resources and how they project themselves in terms of health [35].

All the empirical studies reviewed adopt a social conception of obesity, distanced from theoretical biomedical approaches, which view obesity or being overweight as a disease and analyse it as a risk factor for other diseases. However, within a social conception of the problem, obesity marks a body and affects a person’s social image in terms of first impressions [36]. When adopting a social perspective, the analysis should not only be framed from descriptive research that shows the social groups in which obesity occurs, but should also include the conditioning factors of the actions, the secondary factors that come into play, the consequences for overweight or obese individuals, as well as the cultural resources that they have to deal with the problem [37].

The causal explanations of obesity reviewed here can be grouped into four areas: first come those that link socio-economic variables with obesity, which detect in particular the social groups most affected, according to gender, education, age, or income [14,15,16,17,18,19,20,21,22,23,24,25,26]. Secondly, other studies attribute this problem mainly to the creation of obesogenic contexts and they give special relevance to location as a determining factor in the dietary pattern [27,28]. Thirdly, other studies consider that food choices are explained through the subjective interpretation of reality, emphasising the way in which individuals attribute meanings or interpret food and its characteristics or the effects of ingestion on the body, which guides their choices in the purchase and preparation of food [8,30,31,32]. Fourthly, there are studies that associate eating behaviour and obesity in particular with relationships with other people and the social links established around food [3,29,30,34,35].

This paper asks, as a research question, what the gaps are in official Spanish surveys that hinder analysis of the social determinants of obesity. The aim is to make proposals to improve the instruments in order to facilitate public intervention in the epidemic.

Several researchers have examined the surveys critically in relation to obesity and have raised questions about their limitations. These existing studies consider that measurement needs to be improved: for some, it is necessary to revise the weight and height registers; others suggest a need to modify the list of foods that are asked about; all of them call for agreement between professionals to reach a consensus on the methods for measuring overweight and obesity. Furthermore, the three existing studies coincide in adopting a nutritional approach [38,39,40]. There are no other studies in Spain that address the measurement of social factors influencing obesity, despite the general consensus that these are the main determinants of the increase in overweight and obesity and that, therefore, intervening here is what can help to curb their rise.

The contribution of the present work is the consideration of sources that do not explore strictly nutritional factors but can be used to capture changes in diet over time. Hence, not only is attention paid to the outcome (obesity), but the processes leading to it are included (dietary change) and thus the social determinants of overweight and obesity.

## 2. Materials and Methods

To describe and explain dietary changes, the official European statistical agencies use three surveys, which in the case of the Spanish Statistical Office (hereafter INE) are: the Spanish Health Survey (hereafter ENS until 2006, and ENSE from 2011–2012 onwards) [41]; the Household Budget Survey (hereafter EPF) [42]; and the Time Use Survey (hereafter EET) [43]. All three are standardised with the European surveys of the European statistical office EUROSTAT [44]. None of them were designed to examine food alone, but all are the reference resources for national and European research in this area.

There are two other Spanish surveys that study food that are conducted somewhat irregularly: the Food Consumption Panel of the Ministry of Agriculture [45] and the Aladdin Study on childhood obesity promoted by the Ministry of Health, which has now been conducted four times (2011, 2013, 2015, and 2019) [46]. Unlike the three mentioned above, these surveys are not integrated into the National Statistical Plan (Royal Decree-Law410/2016), so the agencies responsible are not obliged to make the data they collect publicly available and the databases are not public either. Nor do they have direct equivalents in other European countries to enable comparison, as with the statistics of the s Spanish Statistical Office, which we analyse below.

In order to carry out the analysis of the three INE surveys, their methodological files have each been examined and three areas have been analysed: the questionnaires’ objectives, their design, and their variables, in terms of the concepts behind them and their characteristics [47,48,49,50,51,52,53,54].

The ENS National Health Survey has a section on ‘Social determinants’ which asks about food. It enables researchers to describe eating habits based on the frequency of consumption of a short list of foods. It also asks about weight and height, and includes some data to calculate how autonomous the elderly are in their own food care (buying, preparing, and eating). This survey has an extensive list of socio-demographic variables. Its most significant shortcoming is the lack of references to the organisation of daily food and household roles (data on preparation, purchase, and day-to-day organisation), and there is no information on what motivates consumption behaviour.

This survey has undergone some notable changes with respect to our purposes. In the surveys of 2003–2006, weight and height were requested, as in all of them, but only from 2011–2012 onwards was the calculation of the Body Mass Index explained, and this calculation was modified for minors in 2017. In 2003, we started to ask whether the respondent was dieting or followed a special diet, and the reason for this diet. This question was dropped in 2017, when the question about breakfast also disappeared from the adult questionnaire. There are some shifts of interest in the list of foods, with clarifications about the items regularly consumed in 2011–2012, and new categories appear: fast food, pre-meal and savoury snacks, and natural juices.

The EPF provides information on household expenditure. Group 1 is for food and non-alcoholic beverages and Group 11 asks about eating outside the home, currently labelled ‘Restaurants and hotels’. The list of products is extensive and varied and it provides an overview of what is eaten through what is bought for cooking. It has a broad sample base and an exhaustive list of geographical variables, characteristics related to the household and its members. It serves as a basis for preparing important socio-economic indices (calculation of the Consumer Price Index, the list of goods and services that make up the shopping basket). This survey does not show aspects of food related to intake, nor is it linked to health parameters, although a nutritional survey (ENNA-3) directed by Varela Moreira was carried out three times in the 1990s [55]. The survey effectively equates expenditure with consumption and lacks data that would help to understand how this spending is managed within the household. It does not include any variable relating to health.

From 2011–2012 onwards, there have been some changes in the classification of cohabitation and work activity, but these are minimal in the case of spending on food. It is worth noting that in 2016 the ‘Restaurants and hotels’ section included holiday rentals, which further blurs the calculation of food expenditure outside the home.

The EET is oriented towards examining the organisation of time in daily life and has a specific section on how meals and shopping are organised. It records the times of meals, the time devoted to preparation and eating, where meals take place, and the people they are shared with, as well as any other activities engaged in at the same time. It provides information on the incidence of gender roles and inequality in the sharing of tasks both within and outside the home. It does not contain specific records on health, but it registers activities, so it notes if sport is done, if the person goes to the doctor, and any other health-related activity that requires time. Height and weight appear in both iterations.

Some changes occurred between the two iterations. There was a change in the way income was measured, with new bands and slight changes in the variables relating to work activity. The options with respect to occupational status, however, were reduced. The variable for nationality was expanded, asking in 2009–2010 not only whether the respondent was Spanish or foreign-born, but also from the EU or not, and what the country of birth was.

Under marital status, there was a new category of cohabitation (as a couple). The classifications of main and secondary meals were grouped under the same heading and there were slight changes in the classification of tasks involved in preparing meals. More detailed information on the characteristics of the three surveys can be found in the Annex (Supplementary Material Tables S1 and S2).

For a comparative analysis of the three surveys, four criteria have been used, based on the results of the social studies reviewed in the literature review. These studies consider four types of explanatory variables for obesity: those that consider the causes of obesity linked to socio-demographic variables; those that provide contextual explanations and consider variables in location; those that explain obesity on the basis of subjective motivations in the choice and preparation of food; and finally relational variables, associated with the social links between individuals.

## 3. Results

The four criteria used for the analysis of the surveys show the gaps common to all of them, as well as the characteristics that make them complementary. These are summarised in Table 1.

It is, however, not only a question of considering whether the variables are present, but also of specifying in what way they are present, in order to determine their usefulness. To this end, it is possible to observe the way in which these variables are expressed and, in particular, the limitations of each of the surveys (Table 2).

## 4. Discussion

The surveys examined offer information on the complexity of dietary change but do not have enough variables to provide the necessary detail on the social factors linked to obesity.

Socio-demographic variables. The ENS health surveys enable us to correlate obesity with socio-demographic variables, but this is not the case with the other two surveys. The EPF household survey does not provide information on obesity, although it does corroborate dietary inequalities, showing socio-economic differences and the dietary patterns followed by individuals or households according to socio-economic and demographic variables. It provides information on the relationship between economic status and diet through an extensive list of foods. From the EET on time use researchers can extract information about the timing of meals and the importance of different daily food-related activities (preparation and eating) as a function of socio-demographic variables: gender, income, education, and age. It offers information about eating outside the home and also portrays relationships, showing gender roles and social relations in connection with food.

Locational variables. The surveys have some variables associated with the environment, which could help to identify obesogenic contexts. The EET includes classification of province, municipality, and district, and also specifically asks where meals are eaten. The ENS has data by region (Autonomous Community) and considers the population size of communities, allowing differences between urban and rural areas to be captured. The EPF enables researchers to explore variations in food expenditure according to region and by population size or relative concentration. These territorial approaches make it possible to find areas where more or less is spent on fresh or processed products depending on the district (EPF) or the food patterns by region (Autonomous Community). All of this helps to identify variations by area or to determine whether there are food deserts in Spain [56], although only the ENS and the EET allow this information to be associated with obesity. The EPF also makes it possible to differentiate between food eaten at home and out.

Motivational variables. None of the three surveys analysed include any questions aimed at finding out behavioural motivations. It is recognised that healthy habits are helped or hindered by the objective conditions of social life, which allow more or less room for manoeuvre depending on a person’s or family’s social, economic, or cultural capital, in line with the theoretical model proposed by Bourdieu [57]. However, there is no plan for any subjective self-assessment that would provide insight into what incites (or restrains) behaviour, so that it is not possible to know to what extent values, attitudes, beliefs, or the meaning associated with food are related to dietary choices. There are, however, questions about motivation in relation to respondents’ assessment of their own state of health in both the ENS and the EET surveys.

Relational variables. The survey that provides the best information on healthy eating and people’s connections with others is the EET, as eating is associated with the time spent cooking and eating, and the relationship of these activities with other people (when and with whom), both inside and outside the home. The EPF, with its household questionnaires, reveals the collective activity involved in the purchase of food, as well as its quantification in terms of the members of the household. Although the relationships are not so evident in this case, a collective act of spending (and thus consumption) is reflected. These variables are not linked to obesity.

As for the ENS, it has no variables that bring us closer to the social relationships linked to food, except in the case of people over 65 who need care from others. It does, however, allow us to establish relationships between the activities carried out in daily life, such as the consumption of alcohol and tobacco, hours of sleep, or physical exercise, although only the EET confirms the daily routines and how much they fit in with those of other people. All the same, although it is complicated to estimate the importance that an individual or group gives to eating, the EET helps to quantify this through the time spent on eating and cooking.

## 5. Conclusions

It is evident that some of the social factors explaining dietary change can be corroborated with data from the three sources available from the National Institute of Statistics (INE), but this does not imply that it is equally possible to confirm the social factors explaining obesity.

These sources are complementary and their explanatory power would increase substantially with the inclusion of height and weight (for the calculation of the Body Mass Index) within the variables identifying the reporting person and the household members in the EPF.

Its interest is even greater if we bear in mind that this is the survey that incorporates the largest number of foods, which might facilitate the association between social variables and specific food consumption. This would make it possible to detect the relationship between changes in food consumption and the body changes associated with it (overweight and obesity), something of particular interest for analysing periods of crisis where the social context modifies purchasing habits, the effects of which manifest in the medium or long term.

Motivational variables are difficult to fit into a standardised questionnaire, although perhaps the use of Likert scales could facilitate this task without excessive complexity, as is done in national and European opinion barometers. Questions of an evaluative nature about eating habits and reasons for not eating healthily would introduce subjective assessment of interest. This would help substantially in interpreting statistical results, which often only provide figures that are of little use in curbing the epidemic.

The relationships are currently established through the different questionnaires—for the household, for children, and for the individual—and with data on household composition. However, the individualisation of eating leads to the loss of cultural transmission of dietary knowledge. In a context where more and more information is circulating about food in the media and social networks, not knowing how much is known and how people learn to eat and cook means remaining unaware of the reality of social groups with new culinary skills and new consumption practices. Points of reference concerning people’s knowledge about healthy eating and how this knowledge is acquired would provide agencies with valuable information to guide nutritional information for groups at risk of food acculturation.

The incorporation of scales for social relations and motivations related to food in the ENS and the EPF would help to improve these statistical records. These changes would generate synergies that would greatly help to obtain a deeper understanding of the social causes of obesity and would make the current surveys extremely useful.

It is not, therefore, a question of creating new tools for obesity research, but of taking advantage of the capabilities of the INE’s surveys to put rigorous and reliable data into shaping a food policy against a problem whose nature has changed radically.

## Figures and Tables

**Table 1:** Types of variables in the ENS National Health Survey, the EPF Household Budgeting Survey, and the EET Time Use Survey.

	Socio-Economic Variables	Locational Variables	Motivational Variables	Relational Variables	Weight and Height
ENS	X	X	X	X	X
EPF	X	X		X	
EET	X	X	X	X	X

Source: Authors.

**Table 2:** Capabilities and limitations of the National Health Survey (ENS), the Household Budget Survey (EPF), and the Time Use Survey (EET) for capturing the social factors involved in obesity.

	Capabilities	Limitations
**ENS National Health Survey (from INE Methodology 2017)**	Socio-economic and socio-demographic variables Extensive socio-economic information on household members Questionnaire about household, adults, and children Dietary parameters: frequency of food consumption Non-dietary parameters: health status, physical activity, sleep, smoking, alcohol, eating outside the home, sedentary leisure time Subjective perceptions: happiness and health Body mass index (weight and height)	No information on the amount of energy consumed No reference to preparation Lacks questions on motivation, beliefs, and values Lacks questions on nutritional and/or culinary knowledge Does not report on how food is shared in the household Does not report on household roles (who cooks/buys) Does not report on whether or not a diet is followed Self-classification of social class according to occupation Weight and height self-reported
**EPF Household Budget Survey (from INE Methodology 2016)**	Socio-economic and socio-demographic variables, and household characteristics Individual and household questionnaire Links food to the rest of the household budget Extensive list of food products Records quantities purchased Reports on how food is shared within the household	Purchase and consumption are equated Lacks questions on motivation, beliefs, and values Lacks questions on nutritional and/or culinary knowledge Does not report on household roles (who cooks/buys) Lacks variables associated with health and Body Mass Index (weight and height)
**EET Time Use Survey (from INE Methodology 2011)**	Socio-demographic and socio-economic variables relating to the individual and the household Reports on perceived health status Reports on physical activity Reports on activities associated with food and eating Reports on routines and their interaction Reports on social relationships connected with eating Calculates time spent on paid work and household work Reports on household roles (shopping, preparation, and eating) Shows social roles, eating and shopping routines, eating places	Individual questionnaire Does not include food eaten or bought Lacks questions on motivation, beliefs, and values Lacks questions on nutritional and/or culinary knowledge Lacks variables associated with health and Body Mass Index (weight and height).

Source: authors.

## Data Availability

Spanish Statistical Office (INE). Encuesta de Presupuestos Familiares. Available online: https://www.ine.es/metodologia/t25/t2530p458.pdf (Accessed on 23 November 2021). Spanish Statistical Office (INE). Encuesta de Empleo del Tiempo. Available online: https://www.ine.es/dyngs/INEbase/es/operacion.htm?c=Estadistica_C&cid=1254736176815&menu=metodologia&idp=1254735976608 (Accessed on 23 November 2021). Spanish Statistical Office (INE). Encuesta Nacional de Salud. Available online: https://www.ine.es/dyngs/INEbase/es/operacion.htm?c=Estadistica_C&cid=1254736176783&menu=metodologia&idp=1254735573175 (Accessed on 23 November 2021).

## Supplementary Materials

The following supporting information can be downloaded at: https://www.mdpi.com/article/10.3390/ijerph191811156/s1, Table S1: Principal methodological characteristics of the ENS, EPF, and EET surveys, Table S2: General modifications and, in detail, those related to food and eating in the ENS, EPF, and EET surveys in their different versions. References [48,49,50,51,52,53,54,55] are cited in the supplementary materials.
