# Supplementary material for: Are Spanish Surveys Ready to Detect the Social Factors of Obesity?
Source: Int J Environ Res Public Health. 2022 Sep 6;19(18):11156. doi: 10.3390/ijerph191811156 (PMC9517615; doi:10.3390/ijerph191811156)
Supplement: Supplementary file 1 [file ijerph-19-11156-s001.zip › ijerph-1859667-supplementary.pdf]

Table S1. Principal methodological characteristics of the ENS, EPF, and EET surveys.

	**Survey Started**	**The Survey's Principal Methodological Characteristics**
National Health Survey<br>*Encuesta Nacional de Salud*<br>(ENS)	1987	Reference period: 2017.<br>Type of survey: Conducted every 5 years.<br>Population surveyed: Persons living in family homes.<br>Geographical scope: All of Spain.<br>Reference period covered: Last 12 months/4 weeks/2 weeks preceding interview.<br>Sample size: 37,500 households distributed over 2,500 census districts.<br>Type of sampling: Stratified three-stage sampling.<br>Collection method: Computer-assisted personal interview.<br>Questionnaires: Three—household, adult, minors.<br>Baseline quality control: Pretest in the 2006 ENS.
Family Budget Survey<br>*Encuesta de Presupuestos Familiares* (EPF)	1958<br>Two types:<br>- Every eight to ten years (structural)<br>- Quarterly report<br>- In 1997 the two types were combined<br>From 2006, conducted annually	Type of survey: Annual.<br>Population surveyed: Private households.<br>Geographical scope: All of Spain.<br>Reference period covered in results: Calendar year.<br>Reference period covered for information: Varies according to type of expenditure<br>Sample size: 24,000 households (approx.).<br>Type of sampling: Two-stage, with stratification of the units at the first stage.<br>Collection method: Mixed, with direct recording by household and interviews.<br>Questionnaires: Household file; household account books; individual account books; monthly, quarterly, and annual expenses; housing characteristics; and periodic bill and installment payments.
Time Use Survey<br>*Encuesta Empleo del Tiempo*<br>(EET)	1996—first pilot	Type of survey: Variable.<br>Population surveyed: Private households living in main family home, and all individual members of these households.<br>Geographical scope: All of Spain.<br>Reference period covered in results: 2009–11.<br>Sample size: 11,538 households in 1,275 districts.<br>Type of sampling: Two-stage, with stratification of the units at the first stage.<br>Collection method: Filling in individual questionnaire and keeping a diary of activities.<br>Questionnaires: Household questionnaire, individual questionnaire, diary of activities (each person and every day).

Source: Created by authors from information provided by INE on the surveys (www.ine.es) and the methodological data provided in each survey [48–55].

Table S2. General modifications and, in detail, those related to food and eating in the ENS, EPF, and EET surveys in their different versions.

Survey	Methodological Changes
National Health Survey *Encuesta Nacional de Salud* (ENS)	**Methodological Report 2003**<br>Physical characteristics: Weight and height collected.<br>Nutrition:<br>- Frequency of food consumption: Fresh fruit; Meat; Eggs; Fish; Pasta, rice, and potatoes; Bread, cereals; Vegetables; Pulses; Sausage and cold cuts; Dairy products; Sweets.<br>- Type of breakfast: Coffee, milk, tea, chocolate, cocoa, yogurt, etc.; Bread, toast, cookies, cereals, pastries, with or without butter, oil, etc.; Fruit, juice, etc.; Eggs, cheese, cold cuts, bacon, sausages, etc.; Other type of food and/or beverages; Nothing, does not usually eat breakfast.
	**Methodological Report 2006**<br>- Adjustment of the classification of educational level to bring it in line with the National Classification of Education 2000 (CNED2000).<br>- The variable 'country of nationality' is included and the nationality variable is simplified to the answers 'Spanish', 'foreign'.<br>- Simplification of the options for the variable 'relation to economic activity'.<br>Physical characteristics:<br>- Weight and height and the relationship between the two measurements are collected.<br>Food:<br>- Frequency of food consumption: The item 'soft drinks with sugar' is included.<br>- Question on dieting or special diet: To lose weight; To maintain current weight; To live more healthily; Due to illness or health problem; For another reason.
	**Methodological Report 2011–2012**<br>Change of acronym from ENS to ENSE.<br>- Social class is included in the classification variables calculated according to occupation.<br>- In the 'nationality' and 'country of birth' variables, if 'foreign' is answered, the country of origin is asked.<br>- Within the variable 'Situation regarding economic activity' the answer 'working but on sick leave of three months or more' is removed and included under 'working'.<br>Physical characteristics:<br>- Record of details relating to self-reported weight and height and the relationship between the two measures.<br>Definitions:<br>- Body Mass Index (BMI): the ratio of the individual's weight (expressed in kilograms) to the square of the height (expressed in metres).<br>- In the population aged 18 and over: Underweight if BMI < 18.5 kg/m^2; Normal weight if 18.5 kg/m^2 $>$ BMI < 25 kg/m^2; Overweight if 25 kg/m^2 $>$ BMI < 30 kg/m^2; Obese if BMI > 30 kg/m^2.<br>- A special definition of overweight and obesity is used for the population aged 2–17.

	Diet: frequency of food consumption:<br>- Items for fresh fruit, meat, dairy products and sweets are specified: Fruit (except juices); Meat (chicken, beef, pork, lamb etc.); Dairy products (milk, cheese, yogurt); Sweets (cookies, pastries, jams, cereals with sugar, sweets, etc.).<br>- New items included: Fast food (fried chicken, sandwiches, pizzas, hamburgers); pre-meal or salty snack foods (potato chips, cheesy chips, crackers); Natural fruit or vegetable juice.
	**Methodological Report 2017.**<br>- Changes in the classification of educational level to bring it in line with the 2014 National Classification of Education in levels of education attained (CNED14-A).<br>- Minor changes in the classification of the variable 'Status regarding economic activity'.<br>Physical characteristics:<br>- Modification of BMI definitions for children aged 2-17 for underweight, overweight, and obesity.<br>Diet:<br>- Removal of the question on diet and breakfast in the adult questionnaire.
Household Budget Survey *Encuesta de Presupuestos Familiares* (EPF)	**Methodological Report 2006–2015**<br>- From 2011 onwards, a variable has been added within the group 'Characteristics relating to household members'.<br>- Type of union with the spouse or partner: Marriage, Registered unmarried partner, Unregistered unmarried partner.<br>- Modified the variable 'Status of household members regarding economic activity in the week preceding the interview (Monday to Sunday)'.<br>- In the section 'out of work', a difference is made between 'with permanent incapacity to work' and 'person in another situation (not carrying out any economic activity)'.<br>- The variable 'type of working hours: full, part-time' is included.<br>Food:<br>The 12 large expenditure groups: 1. Food and non-alcoholic beverages; 2. Alcoholic beverages, tobacco, and narcotics; 3. Articles of clothing and footwear; 4. Housing, water, electricity, gas, and other fuels; 5. Furniture, household equipment, and running costs of the dwelling; 6. Health; 7. Transport; 8. Communications; 9. Leisure, entertainment, and culture; 10. Education; 11. Hotels, cafés, and restaurants; 12. Other goods and services.
	**Methodological Report 2016**<br>Changes in the nomenclature of some of the 12 major expenditure groups:<br>- 2. Alcoholic beverages and tobacco; 3. Clothing and footwear; 5. Furniture, household goods and articles for running household; 6. Health; 7. Transport; 9. Leisure and culture; 11. Restaurants and hotels.<br>- Changes in the items included in each category of expenditure; the most important one in relation to food is the inclusion of holiday rentals under 'restaurants and hotels'.
Time Use Survey	**Methodological Report 2002–2003**<br>Average net monthly income of the household: Under €1,000; From €1,000 to €1,499.99; From €1,500 to €1,999.99; €2,000 and over.

Encuesta de Empleo del Tiempo (EET)	Food and drink<br>- Main meals and drinks<br>- Non-main meals and beverages<br>- Non-main meals and beverages not specified<br>- Snacks, tapas<br>- Snacks<br>- Other non-main meals and beverages<br>Cooking activities:<br>- Meal preparation<br>- Baking<br>- Dishwashing<br>- Food preservation<br>- Other specified cooking activities
	**Methodological Report 2009–2010**<br>- Modified details under the variable 'level of education attained'.<br>- Removed the variable 'chronic illness'.<br>- Modified the variable 'nationality', including the variable 'country of birth'.<br>- Included 'Living together as a couple'.<br>- Removed variable 'employee's sector of activity'.<br>- Simplified the categories in the classification variables for dependants.<br>- Included variable 'Children under 18 in another household'.<br>- Removed the following variables related to housing: 'type of dwelling occupied' and 'type of tenancy for the dwelling occupied'.<br>- Modified the ranges for regular monthly net income of all household members.<br>Food:<br>- Modified the section that differentiated main meals from non-main meals, including all in the same category.<br>- Under cooking activities, the item 'clearing the table' is altered to 'Clearing the table after breakfast, lunch, dinner, snack, coffee...'.

Source: Created by authors from the methodological reports in the each surveys [48–55].
